# A Novel Solution for Distal Dilation of Chronic Dissection After Repair Involving Visceral Branches: The Road Block Strategy

**DOI:** 10.3389/fcvm.2022.821260

**Published:** 2022-03-09

**Authors:** Yiming Li, Zhenjiang Li, Jiaxuan Feng, Rui Feng, Jian Zhou, Zaiping Jing

**Affiliations:** ^1^Department of Cardiology, General Hospital of Northern Theater Command, Shenyang, China; ^2^Endovascular Diagnosis and Treatment Center for Aortic Diseases, Department of Vascular Surgery, Changhai Hospital, Navy Medical University, Shanghai, China; ^3^Department of Vascular Surgery, The First Affiliated Hospital of the Medical School of Zhejiang University, Hangzhou, China; ^4^Department of Vascular Surgery, Shanghai General Hospital, Affiliated to Shanghai Jiaotong University, Shanghai, China

**Keywords:** aortic dissection (AD), novel procedure, endovascular treatment, vascular remodeling, thrombosis

## Abstract

**Aim:**

Notwithstanding that unprecedented endovascular progress has been achieved in recent years, it remains unclear what is the best strategy to preserve the blood perfusion of abdominal visceral arteries and promote positive aortic remodeling in patients with distal dilatation of chronic aortic dissection in abdominal visceral part (CADAV) after aortic repair. The present study developed a Road Block Strategy (RBS) to solve this conundrum.

**Methods and Results:**

This prospective single-center clinical study included patients suffering from symptomatic distal dilatation of CADAV after aortic repair treated with RBS from January 2015 to December 2019 and followed up regularly for at least 2 years. Stent grafts were implanted first to cover distal tears and expand the true lumen. Device embolization was performed to induce proximal and distal segmental false lumen thrombosis (FLT) apart from the level of the ostia of vital branches. Successful RBS was performed in 13 patients. Significant differences were found in maximum true lumen diameter (*p* < 0.05), blood flow area in false lumen (FL) (*p* < 0.001), and the ratio of blood lumen to FL area (*p* < 0.05) between the pre-procedure and the latest follow-up results. No aortic rupture, vital branches occlusion, thoracic and abdominal pain, or death occurred during hospitalization and follow-up.

**Conclusions:**

Our findings suggest that RBS is feasible in treating distal dilatation of chronic aortic dissection after prior proximal repair, inducing false lumen thrombosis, preventing deterioration of aortic dissection, and maintaining the patency of abdominal visceral arteries.

## Introduction

Aortic dissection is one of the most dangerous vascular diseases (1, 2), given that it can cause an aortic rupture in a short period leading to death (3, 4). Thoracic endovascular aortic repair (TEVAR) has been regarded as first-line therapy for complicated Stanford type B aortic dissection and a pre-emptive measure to avoid late complications by inducing aortic remodeling (5, 6).

However, complications after TEVAR seriously affect aortic dissection's prognosis, most of which need to be resolved. Distal dilatation is one of the common complications of chronic aortic dissection in the visceral part (CADAV) after proximal TEVAR, often involving the visceral artery arising from the sac or false lumen (FL) (7). Distal dilatation has been reported in one-third of chronic aortic dissection patients after proximal entry tears exclusion (8). Moreover, regression analysis showed that visceral branches arising from the FL represent an independent risk factor [odds ratio (OR) = 10.1] for negative abdominal aortic remodeling (9). Currently, available techniques still bear significant limitations. Covered stents cannot simply exclude all the tears, multilayer stents do not reduce the blood pressure in the FL, the risk of endoleak is still present with the fenestration technique and the chimney graft tends to be occluded (7, 10).

The state of the FL plays an important role in the incidence of complications of aortic dissection after TEVAR; complete thrombosis of the FL has been associated with a good prognosis (11), while patients with a patent FL have an increased risk of aortic expansion and death (3, 12). Partial thrombosis of the FL has been established as a significant predictor of mortality during follow-up (13). Therefore, complete FL thrombosis (FLT) is an important object of endovascular procedures in the follow-up period (14, 15). Moreover, a series of FL embolism studies showed that FL could also be treated with embolization devices such as plug, coil, Onyx glue, and occlude (16–19). In the present study, we documented a novel therapeutic approach for promoting FLT *via* currently available embolization devices to preserve blood flow of the abdominal visceral artery, reduce blood flow in the FL to improve patient outcomes. Given that the embolus in the FL obstructs blood flow in any direction except for the visceral artery and the shape and functions are similar to roadblocks, this approach was termed as Road Block Strategy (RBS).

## Materials and Methods

### Patients

The experimental protocol and all procedures performed in this study were in accordance with the Ethical Standards of the Biomedical Research Ethics Committee of the Second Military Medical University (Navy Medical University) (protocol number SMMU-8182500272) and with the 1964 Helsinki Declaration and its later amendments or comparable ethical standards. All patients enrolled in the study provided informed consent for the procedure. From January 2015 to December 2019, 139 patients with chronic aortic dissection whose abdominal visceral arteries were supplied or partially supplied by a FL documented by computed tomographic angiography (CTA) were treated in our center. The inclusion criteria were (1) Period between surgery and aortic dissection onset >90 days; (2) At least one visceral artery is supplied by FL; (2) Having symptoms associated with aortic dissection recently or maximum diameter of abdominal aorta >45 mm, or maximum diameter of abdominal aorta growth rate >5 mm per year, confirmed by CTA; and (4) Proximal aortic dissection had been treated by open surgery, hybrid surgery, or endovascular repair.

**Figure 1:**
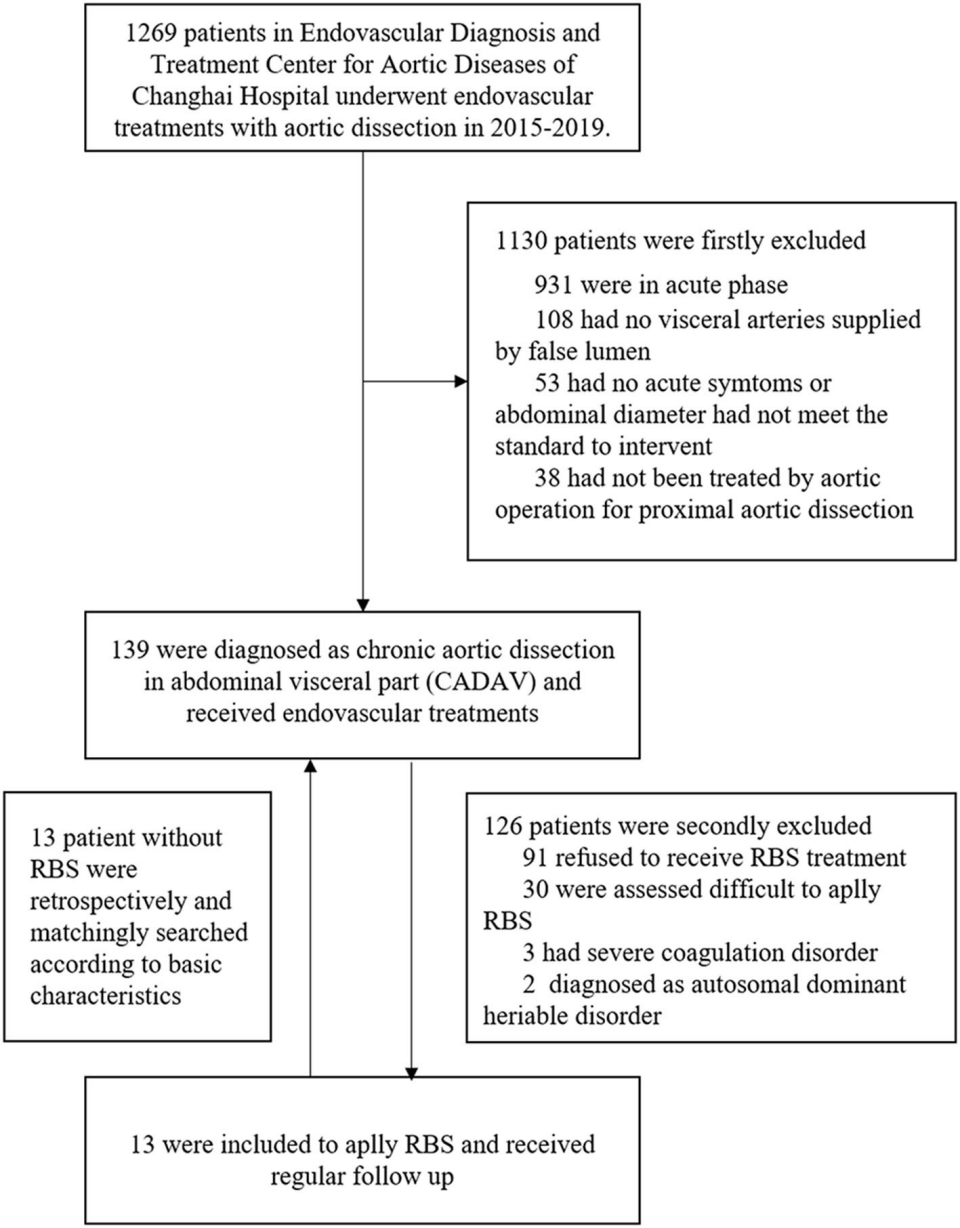
Study population and inclusion/exclusion details.

The exclusion criteria consisted of (1) Patients that refused to receive RBS procedure; (2) Patients that assessed difficult to apply RBS, such as distal tears were excluded or too small to let guidewire pass through; (3) Presence of any coagulation disorder, such as severe thrombocytopenia or hemophilia; (4) Patients diagnosed with autosomal dominant heriable disorder, such as Marfan syndrome. After inclusion and exclusion, 13 patients finally included to apply RBS. According to their basic characteristics, anatomical features and previous solutions of aortic dissection, another 13 patients without RBS were retrospectively matched for comparation (Figure 1).

### Preoperative Management and Evaluation

Drugs such as diltiazem, urapidil, and nicardipine were infused through peripheral veins; nifedipine, valsartan, and metoprolol were taken orally to maintain normal blood pressure and heart rate before the intervention (20). Analgesics and sedatives were given whenever necessary. All patients underwent a CTA scan for aortic remodeling evaluation. Measurements including the maximum diameters of the TL, FL, and total aorta, areas of TL, FL, and blood flow in the FL at the level of the largest diameter of the total aorta, were performed by two independent physicians (YL and ZL), and disparency were judged by the third physician (JZ). All CTA images underwent three-dimensional reconstruction using Aquarius Workstation (TeraRecon, California, USA). The status of the FL in the abdominal visceral part was assessed using the delayed phase images.

### Road Block Strategy

#### Stent Plantation

The first step of the procedure was to place stents in the true lumen guided by digital subtraction angiography (DSA). Given that the proximal tears of the aortic dissection were already excluded, we then evaluated for endoleaks and the need for stenting. To manage tears distal to the visceral arteries (distance between them >2 cm), a stent/stents was/were used to cover these tears and reconstruct the TL. The procedure was performed under local anesthesia. The covered stent graft was advanced into the aorta over a super-stiff guidewire. With the outer sheath remaining in the aorta, the stent graft within the soft sheath was advanced to the distal tear. The soft sheath was removed once the stent graft was delivered proximal to the tears. As for distal tears near visceral arteries (distance between them <2 cm), bare stents were used to enlarge the TL while preserving blood support to the branches. The stent graft was deployed by withdrawing the trigger wire. Immediate angiography was conducted to assess whether the target tear was excluded and visceral arteries were patent.

#### Embolization of the False Lumen

The second step of the procedure was to embolize the whole FL except the level of the visceral artery ostia. A 0.035-inch guidewire was advanced to the FL through a distal unexcluded tear in the abdominal visceral segment. Next, detachable coils (Boston Scientific, Massachusetts, USA) were deployed into the FL. After setting the first coil stably in the FL, the remaining coils were gradually deployed from this initial point.

**Figure 2:**
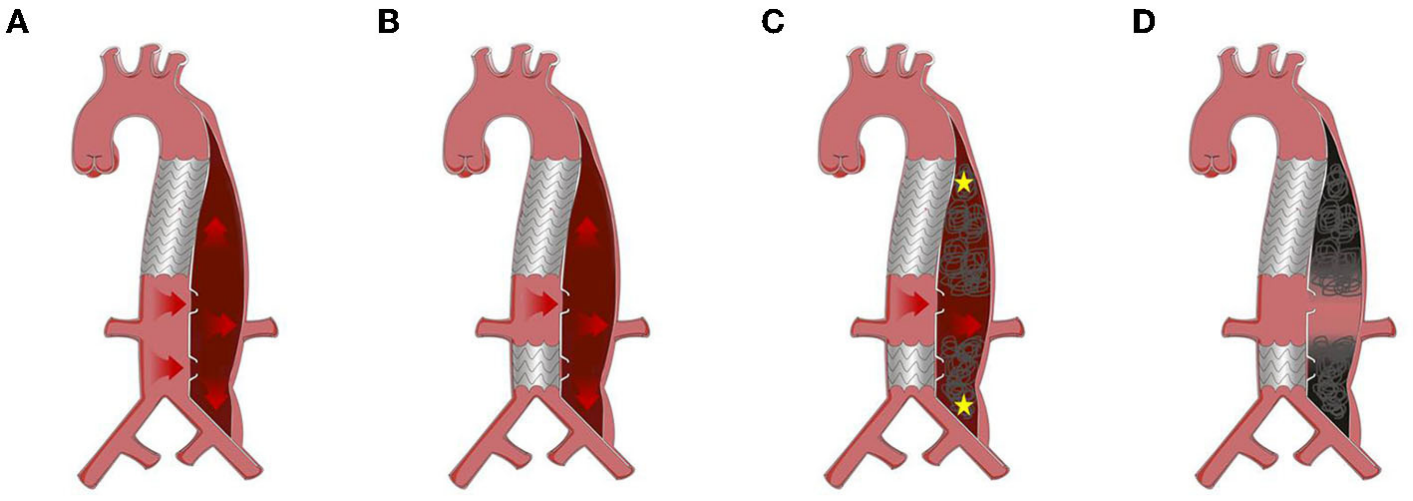
Protocol of road block strategy (RBS). **(A)** Distal dilatation of chronic aortic dissection in the visceral part (CADAV), red arrow represents blood flow direction. **(B)** Excluding tears away from visceral artery with covered stent. **(C)** False lumen (FL) embolism with coils, Onyx glue, or occluders, from the proximal and distal initial point (yellow asterisk) to the level of visceral arteries, preserving the blood flow to perfuse vital branches (red arrow). **(D)** Segmental thrombosis formed in the FL while visceral artery remained patent.

Onyx glue (ev3 Neuroavascular, California, USA) was used to embolize the remnant FL. Occluders (Shanghai Shape Memory Alloy, Shanghai, China) were applied in the FL when FL was too large to be embolized by coils or glue (minimum diameter of FL >20 mm). The procedure was stopped when the angiography showed no blood flow in the FL except at the level of the abdominal visceral artery. Figure 2 shows the entire process of the RBS for treating a patient with distal dilatation of CADAV.

## Follow-Up

Computed tomographic angiography was performed before operation, at 12 months and annually after operation (21). The thrombosis status in the FL, the morphology of the total aorta, unexcluded distal tears, and patency of the visceral arteries were evaluated. The maximum diameters and area of the FL, true lumen and the total aorta were also recorded. Emergency CTA was performed if patients presented with signs or symptoms of aortic complications. All evaluations were performed by at least one vascular surgeon and one radiologist independently.

**Figure 3:**
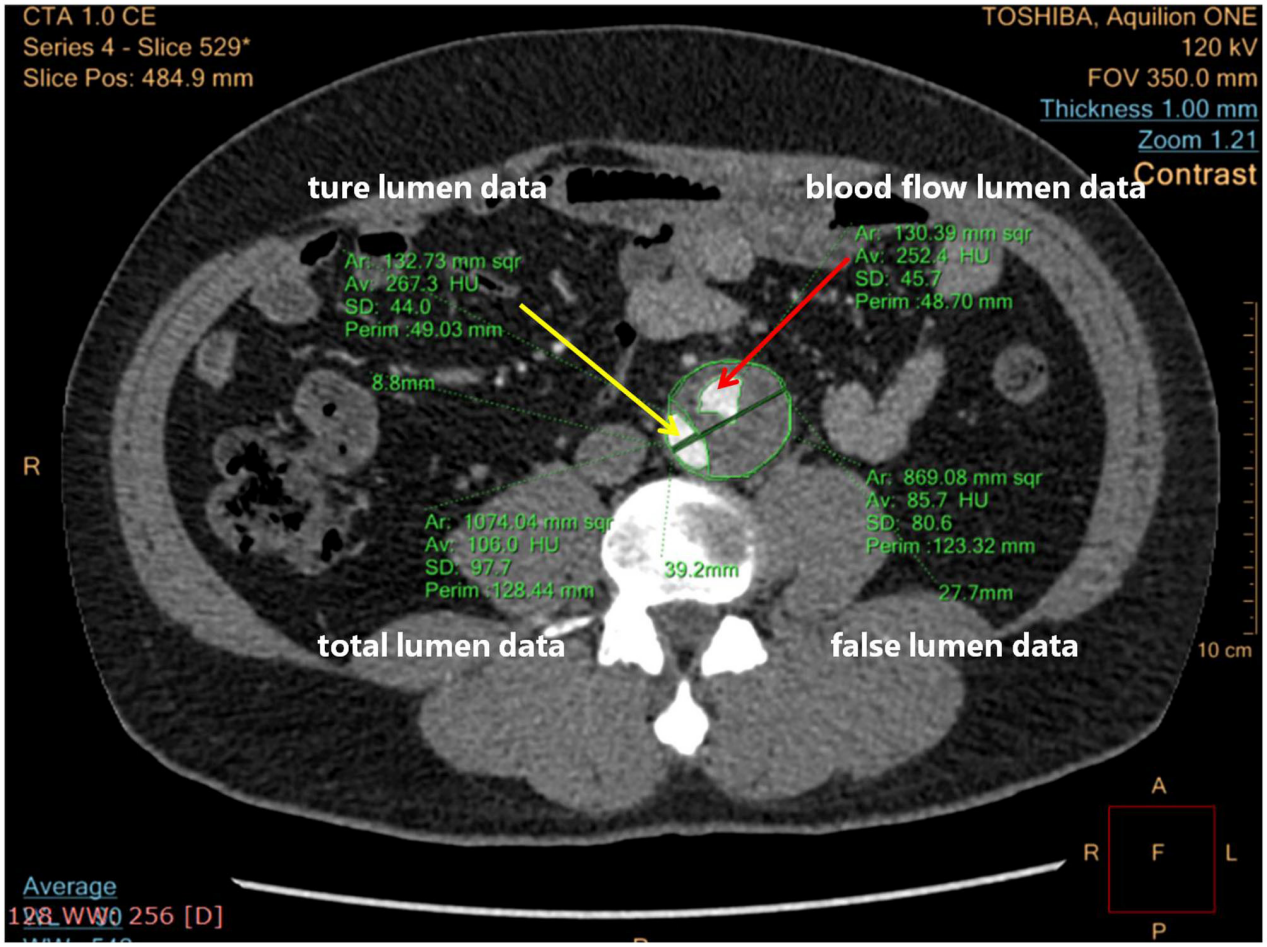
Computed tomographic angiography (CTA) cross-section measuring details blood flow lumen. The yellow arrow points to the true lumen; the red arrow points to the blood flow lumen.

The maximum intensity projection (MIP) mode of CTA was used to show how endovascular devices were used, and three-dimensional reconstructions were used for remodeling of the aorta (22). The FLT status was assessed in two ways: blood flow area in FL, or ratio between it and FL area, especially at the level of the maximum aortic dilatation. Figure 3 shows how the total aortic lumen, true lumen, FL, and blood flow lumen were measured. Blood flow lumen means the cavity of flowing blood the in FL. The blood flow areas mentioned above were measured by software analysis to outline the contour of the blood flow, reading the area, and summing them.

## Statistical Analysis

Data included dimensions of the total aorta, true lumen, FL, and blood flow in FL were measured before and after the induction of FLT, performed as previously described (23). After testing all data for normal distribution and homogeneity of variance, paired *t*-tests, and Wilcoxon tests were performed for parametric and non-parametric data, respectively. All data analyses were performed using SPSS^®^ Statistics 21 (IBM Corp, New York, USA).

## Results

A total of 139 patients were diagnosed with chronic aortic dissection involving visceral arteries at our center from January 2015 to December 2019. A total of 126 patients were excluded for the following reasons: 91 refused to receive RBS treatment, 30 were assessed difficult to apply RBS, 3 had severe coagulation disorder, 2 diagnosed as autosomal dominant heritable disorder. In order to compare the effectiveness of RBS, we retrospectively selected 1:1 paired matched patients (*n* = 13) who had not underwent RBS and followed up regularly in the 126 excluded patients. Finally, a total of 13 patients suffering from distal dilatation of CADAV who underwent RBS and followed up regularly were included in the present analysis.

The demographic and pathological data for these 13 RBS patients and their 13 paired matched non-RBS patients are presented in Table 1. The mean age was 53.8 ± 13.1 years old for RBS patients and 55.6 ± 12.6 years for non-RBS. All patients were diagnosed with CADAV and had indications for re-intervention, as their FL expansion was observed in the abdominal visceral region or they had acute symtoms again since previous aortic repair until RBS. Their previous therapies were strictly controled; therefore, the comorbidities were hard to limit to the same. All patients previously underwent aortic repair, and 5 patients in each group presented distal dissection in the visceral segment previously been treated by endovascular techniques (bare stent or branch stent plantation).

**Table 1:** Patients' baseline characteristics.

**Case number**	**Sex**	**Age**	**Comorbidities**	**Acute symptoms**	**Previous therapies**	
					**Proximal tears therapy**	**Distal tears therapy**
1	M	46	HP	Abdominal pain	Hybrid operation	Palliative care
1*	M	44	HP	Abdominal pain	Hybrid operation	Palliative care
2	M	64	HP	Chest pain	TEVAR	Palliative care
2*	M	62	HP	Lumbago	TEVAR	Palliative care
3	M	35	HP	Abdominal pain	TEVAR	Palliative care
3*	M	37	HP	No	TEVAR	Palliative care
4	F	54	HP & diabetes	Chest tightness	TEVAR	Iliac artery stenting
4*	M	56	HP	Abdominal pain	TEVAR	Iliac artery stenting
5	M	59	HP	Abdominal pain	TEVAR	Palliative care
5*	M	59	HP	Abdominal pain	TEVAR	Palliative care
6	M	61	No	Chest tightness	TEVAR	Palliative care
6*	M	64	No	Abdominal pain	TEVAR	Palliative care
7	M	39	HP	Chest pain	TEVAR	Iliac artery stenting
7*	M	42	HP	Abdominal pain	TEVAR	Iliac artery stenting
8	M	42	HP	Chest pain	TEVAR	Bare stent
8*	M	45	HP	Back pain	TEVAR	Bare stent
9	M	79	Coronary disease	Abdominal pain	EVAR	Palliative care
9*	M	79	HP	no	EVAR	Palliative care
10	M	71	HP	Chest pain	TEVAR	Palliative care
10*	M	73	No	Back pain	TEVAR	Palliative care
11	F	52	No	Abdominal pain	TEVAR	Bare stent
11*	M	55	No	Abdominal pain	TEVAR	Bare stent
12	F	57	HP & coronary disease	Abdominal pain	TEVAR	Bare stent
12*	F	62	HP	Abdominal pain	TEVAR	Bare stent
13	M	41	Pancreatitis	Chest pain	TEVAR	Palliative care
13*	M	45	Coronary disease	Chest pain	TEVAR	Palliative care

* *Patients in the control group with CADV are matched according to the basic characteriscs and previous treatments, whose visceral arteries were not treated by RBS; HP, hypertension; TEVAR, throracic endovascular aortic repair; EVAR, endovascular aortic repair especially for aorta below the diaphragm*.

Details of the two-step procedure for RBS are presented in Table 2. The treatments of non-RBS patients are not listed in Table 2 for their visceral region were not treated additionally and all of their treatments have been listed in Table 1. At least one visceral artery (celiac trunk, superior mesenteric artery, inferior mesenteric artery, or renal arteries) was supplied by the FL in all RBS or non-RBS patients. During the first step of RBS, covered stents were used in 8 patients (62%), bare stents in 3 patients (23%), and no stents were used to maintain visceral artery perfusion in the remaining 3 patients with only the initial tear preserved.

**Table 2:** RBS details in all period.

**Case number**	**Follow-up period**	**Preoperative status**	**Intraoperative status**	**Postoperative status**		
		**Visceral artery involvement**	**RBS details**	**Immediate situation after intervention**	**Complications**	**Involved visceral artery status**
1	31	Left renal artery	2 covered stents & 6 coils & 1 ml glue	No endoleak	No	Dissection, patent
2	43	Left renal artery	6 covered stents & 18 coils & 5 ml glue & 2 occluders	No endoleak	No	Metal shadow, distally patent
3	12	Right renal artery	2 covered stents & 12 coils	No endoleak	No	Patent
4	24	Right renal artery	2 covered stents and 1 bare stent & 8 coils	No endoleak	No	Metal shadow, distally patent
5	13	Left renal artery & inferior mesenteric artery	3 covered stents & 8 coils	No endoleak	No	Metal shadow, distally patent
6	21	Celiac trunk	3 bare stents & 8 coils	No endoleak	No	Metal shadow, distally patent
7	28	Superior mesenteric artery	5 coils & 3 ml glue	No endoleak	No	Metal shadow, distally patent
8	18	Inferior mesenteric artery	1 covered stent & 2 coils	Endoleak	No	Metal shadow, distally patent
9	37	Inferior mesenteric artery	7 coils	No endoleak	Endoleak	Narrow
10	25	Celiac trunk	5 coils	Endoleak	Endoleak	Patent
11	12	Right renal artery	1 bare stent & 8 coils & 3 ml glue & 1 occluder	No endoleak	No	Metal Shadow, distally patent
12	34	Celiac trunk & right renal artery	2 covered stents & 10 coils & 2 occluders	Endoleak	Endoleak	metal shadow, distally patent
13	12	Celiac trunk	1 covered stent & 3 coils	Endoleak	Endoleak	Dissection, patent

*Visceral artery involvement, visceral artery which was supported by FL; RBS, the Road Block Strategy; endoleak, there was blood flow remaining in FL except the tract between the necessary tear and the involved visceral artery; metal shadow, hyper signal of stent, coil or occluder appeared on CTA image*.

**Figure 4:**
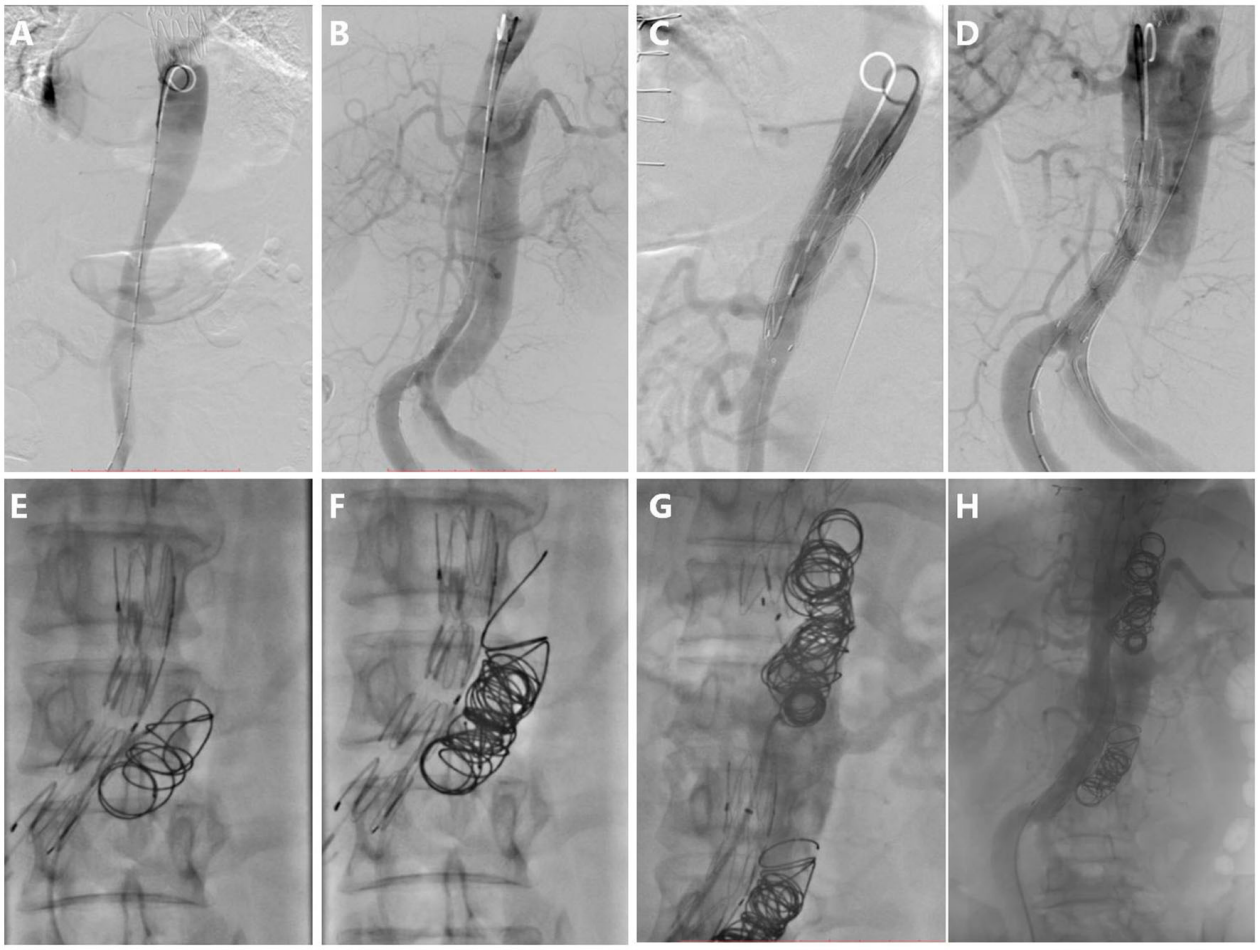
Intraoperative angiography. **(A)** Digital subtraction angiography in the true lumen, **(B)** Aortography in the FL, **(C)** Stent deploying at tears distal to visceral artery, **(D)** Preserving tear which is proximal and supplies blood to the visceral artery, **(E)** The first coil was released and drifted to the initial point in the FL, **(F)** More coils were released from the initial point, **(G)** Repeating procedure F and forming segmental thrombosis, and **(H)** Aortography after RBS, showing blood flow decrease in the FL apart from the necessary blood flow track.

For the second step of RBS, 7.7 ± 4.1 (range 2–18) coils were used in each patient. Coils were placed in the true lumen in 5 patients (38%) and the visceral artery in 3 patients (23%) during embolization, and successfully repositioning was observed in all cases. The coils were extended to the rest of the FL; care was taken to avoid excluding visceral arteries. A total of 4 patients (30%) underwent Onyx glue injection (mean volume of glue injected was 0.9 ± 1.7 ml), and 3 patients (23%) underwent occluder placement due to a large FL (minimum FL diameter > 20 mm) (Figure 4). Intraoperative angiography can be seen as Figure 4.

The average follow-up period in RBS patients is 23.85 ± 10.38 months, while that is 26.46 ± 15.55 months in non-RBS patients. Segmental complete thrombosis in the FL was found in 9 patients immediately after RBS, while segmental complete thrombosis formed in 2 patients in non-RBS group and caused one case of left kidney atrophy. Although endoleaks were observed in 2 patients immediately after RBS, blood flow in the FL was limited. Overall, the only complication was endoleak (*n* = 4), and no patient experienced acute sympoms in the follow-up period. As for non-RBS patients, complications include 1 case of aortic rupture, 2 cases of denovo aortic dissection, 1 case of enlargement of aortic dissection involvement, 1 case of left kidney atrophy, and 9 cases of continuous enlargement of aortic lumen. As for RBS patients, no cerebral infarctions, new dissections, and visceral artery stenosis or occlusion were observed during the follow-up.

After RBS, 9 patients experienced abdominal segmental complete thrombosis. Comparison of the preoperative and the latest postoperative data showed significant differences in three indicators of aortic remodeling at the max diameter level of the total aorta. The maximum TL diameter was significantly increased (14.57 to 18.34 mm, *p* ≤ 0.033), while decreases in blood flow area in the FL (1042.59–to 158.76 m^2^, *p* < 0.001) and Rbf (ratio of blood flow area in FL to FL area) (80–18%, *p* = 0.002) (Table 3), were observed. In terms of outcomes of involved visceral arteries, all patients exhibited patent visceral arteries. A total of 12 patients experienced good visceral artery blood perfusion, and 1 patient exhibited mild visceral artery stenosis related to congenital renal artery malformation.

**Table 3:** Impact of RBS on aortic remodeling.

**Variable**	**Preoperation**	**Postoperation**	**Change**	***P*** **Value**
Maximum aortic diameter, mm	44.94	44.65	−0.29	0.87
**Maximum true lumen diameter, mm**	**14.57**	**18.34**	**4.53**	**0.033**
Maximum false lumen diameter, mm	30.48	26.85	−3.63	0.133
Total aortic area, mm^2^	1,693.87	1521.77	−172.1	0.076
True lumen area, mm^2^	274.22	332.39	58.17	0.152
False lumen area, mm^2^	1,307.14	1071.12	−236.02	0.064
**Blood flow area in false lumen, mm** ^**2**^	**1,042.5938**	**158.76**	–**883.83**	**<0.001**
**R**_**bf**_ **, %**	**0.8**	**0.18**	–**0.62**	**0.002**
R_tt_, %	0.19	0.27	0.08	0.55

*Rbf, ratio of blood flow area in FL to FL area; Rtt, ratio of true lumen area to total aortic area. Bold values indicate statistically significant differences (p < 0.05)*.

**Figure 5:**
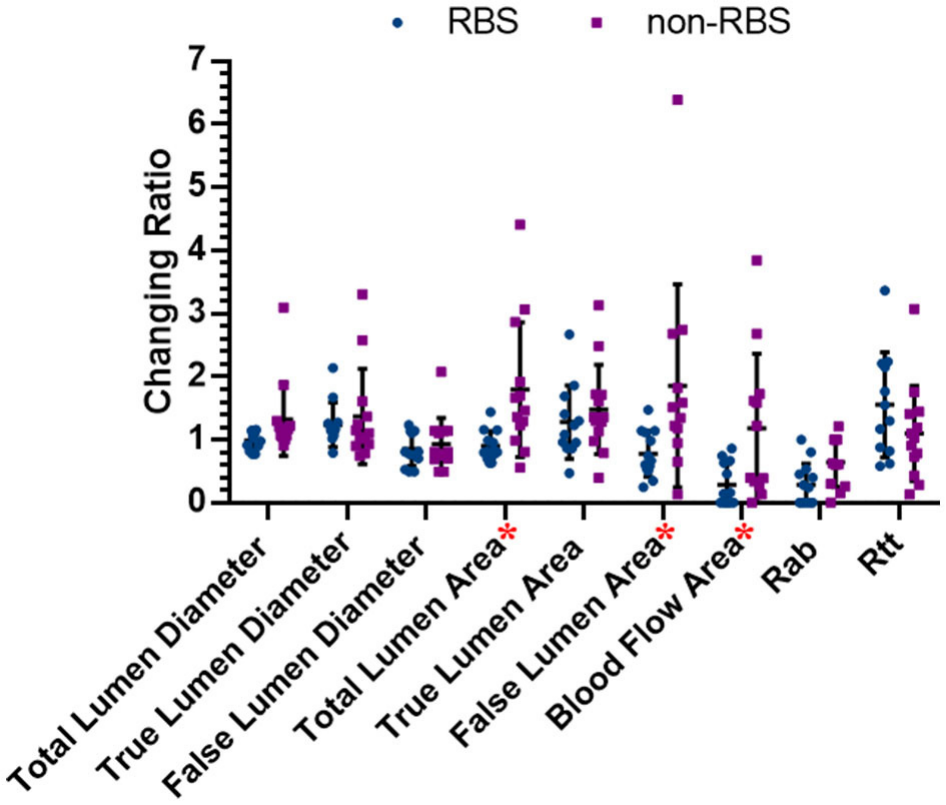
Changing ratios in both RBS and non-RBS groups. Changing ratio: the latest data/preoperative data; * represents there is significant difference between two groups (*p* < 0.05).

Figure 5 shows the comparing results of all the changing ratios between the latest follow-up and preoperative data in two groups. The changing ratio between RBS and paired non-RBS patients in total lumen area (dif = −0.89, *p* = 0.007), FL area (dif = −1.07, *p* = 0.028), and blood flow area (dif = −0.90, *p* = 0.015) shows significant difference, which means patients receving RBS are more likely to decrise the areas of total aortic lumen, true aortic lumen, and blood flow in the FL than those in non-RBS. As for the changing ratio of other items, although the results are not statistically significant, all the changing ratios in RBS group have the positive trend than those in non-RBS group.

**Figure 6:**
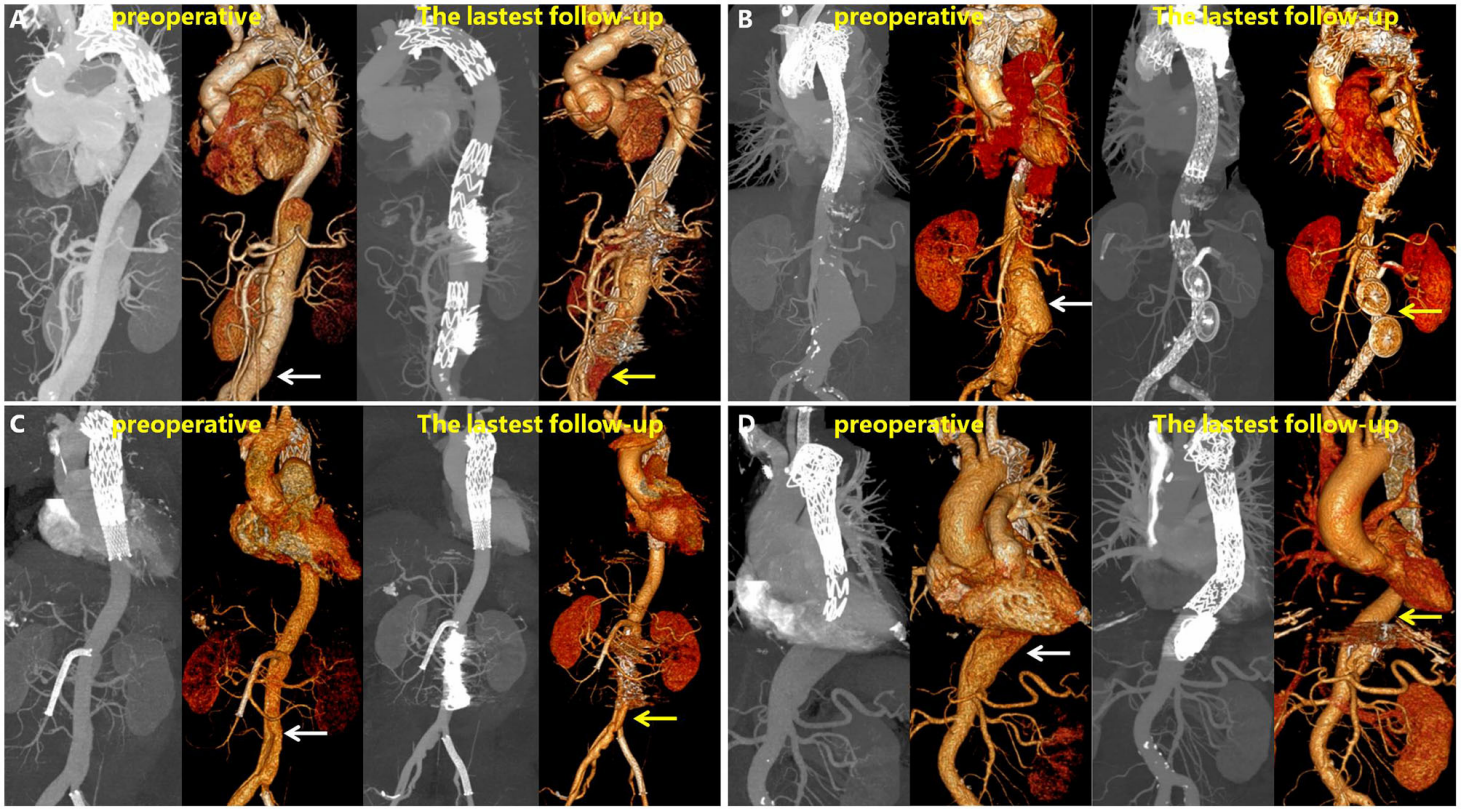
Maximum intensity projection (MIP) and 3D reconstructions of CTA in patients before and after RBS. White arrow points toward dilatation of the FL, and yellow arrow toward thrombosis formation in the FL or shrinking of the FL. **(A)** Patient's left renal artery was involved, and a slight endoleak was observed at 31 months. **(B)** Patient's left renal artery was involved, the FL disappeared at 43 months on 3D reconstruction images. **(C)** Patient's superior mesenteric artery was involved, the FL shrinked at 28 months. **(D)** Patient's celiac trunk was involved, the FL shrinked at 25 months.

Figure 6 shows the representative images of the anatomic changes over time in the MIP mode and three-dimensional reconstruction of CTA before (preoperative) and after RBS (latest follow-up). A video of the three-dimensional reconstructions during the preoperative period and the angiography results before RBS and at the latest follow-up is available in the Supplementary Materials. One typicle case of negative aortic remodeling, denovo aortic dissection and aortic rapture in non-RBS group can be seen in Supplementary Figure 1.

## Discussion

Thoracic endovascular aortic repair was first applied in aortic dissection by Dake and colleagues in 1994 (24). Since then, TEVAR has developed rapidly and is considered the mainstay of treatment for complicated Stanford B aortic dissection (21). Complications after TEVAR limit its use, emphasizing the need to develop new approaches to manage them. Although some stent graft-related complications can be reduced with the significant scientific progress made and new material development, the success of TEVAR is largely limited by complicated aortic dissections, especially in cases where vital branches supplied by the FL need to be preserved. As for aortic dissection involving visceral arteries, TEVAR has been associated with more complications than open repairs during the follow-up period (25). Excluding tears as much as possible by covered stents have been associated with paraplegia and other organ infarction complications (26). As for the Chimney technique, the long-term patency of the branched stents cannot be guaranteed (27–29). Finally, when the fenestration technique is applied for the chronic thoracoabdominal aortic dissection with dilatation in the visceral part, blood support of vital branches cannot be guaranteed (30) and is highly at risk of endoleaks (with a reported incidence of 66.7%) (31).

The above findings highlight the need to develop a novel technique using commercial endovascular devices, which can neither induce complete thrombosis in the FL nor ignore the distal tears. At present, therapies which induce FLT are separated into 2 types. The first type is the common TEVAR treatment, where covered stent grafts with enough radial supporting force are needed. The benefits of the covered stent grafts include restoring blood flow in the true lumen and excluding intimal tears in aortic dissection, often resulting in a high degree of FLT at the stent level in the thoracic aorta (11). The second type involves using coils, Onyx glue, occluders, even “candy-plug” to embolize the FL (16–18).

An increasing body of evidence suggests that using coils along with Onyx glue or occluders is a safe and efficient approach to providing a durable thrombogenic environment (17, 18). During the RBS procedure, these embolization techniques and devices were comprehensively used to induce controlled segmental thrombosis in the FL. The use of embolization devices can mitigate FL blood flow and extend the zone of thrombosis in the FL to create sufficiently organized thrombi that can hardly migrate. The organized thrombi allow blood into the visceral branches and prevent blood flow into the rest of the FL.

Although the prevalence of aortic dissection is not high, aortic dissection-related complications are common in patients that undergo TEVAR, especially in dissections involving visceral arteries. To avoid over-treatment, cases of distal dilatation of CADAV after TEVAR were selected as candidates of RBS. Chronic (from onset to RBS >92 days) aortic dissection was an inclusion criterion of the present study as it is associated with the highest rates of aortic reintervention (39%) compared to acute and subacute aortic dissections (20 and 22%, respectively) (32). Positive aortic remodeling is more prominent in acute aortic dissection than in chronic aortic dissection (33). We fully informed patients about the risks of this novel technique and unknown prospect, patients who hesitated about RBS were regarded as refusing this technique. Moreover, if the FL near distal tears had already happened thrombosis or the distal tears were too small to let guidewire pass through, we still apply non-RBS technique to those patients. Accordingly, above 90% of patients with aortic dissection were excluded to determine which patients truly needed RBS. Although the sample size was small, all screened 13 patients had similar clinical features (dilatation of CADAV, acute symptoms had at least one vital branch supported by the FL).

Clinically, the aortic diameters and FLT status are important indicators for evaluating the aortic remodeling due to their close relationship with complications. The International Registry of Acute Aortic Dissections (IRAD) revealed that an aortic diameter of 5.5 cm was associated with a 4-fold increase in mortality (34, 35). Importantly, a FL diameter larger than 22 mm is predictive of an aneurysm with a sensitivity of 100% and specificity of 76%. Even though the true lumen diameter can change with blood pressure and cardiac cycle, it is still an important predictor of blood support to vital organs and tissues (36, 37). Moreover, complete FLT is the ultimate goal of treatment since it indicates a good prognosis (11). In the present study, RBS induced positive aortic remodeling both in self-control and matched pair comparison. In self-control results, RBS patients received significantly positive differences in maximum true lumen diameter, blood flow area in FL and R_bf_, while receiving significant benefits in areas of total lumen, FL and blood flow in matched pare comparison. Also, complications in RBS are acceptable while those in non-RBS was more fetal and severe. Therefore, RBS has huge clinical application prospects and provides new insights to solve aortic dissection involving visceral arteries, especially zones 6–8 (38).

One major limitation of this study was the small sample size (*n* = 13 *vs*. 13). However, precise induction of FL thrombosis and reversion the trend of dilatation in CADAV are the benefits of this strategy. Further studies with larger sample sizes and advanced RBS are needed to induce more significant positive aortic remodeling and enlarge RBS' indications.

## Conclusion

In distal dilatation of CADAV patients, the unique combination of stenting and embolization devices can induce segmental thrombosis in the FL, solving the conundrum of excluding tears and preserving blood perfusion of the involved visceral arteries. The short-term outcomes of the patients in the present study were satisfactory. Accordingly, inducing segmental thrombosis in visceral arteries perfused by the FL is a safe, effective, and repeatable approach to preserve blood flow and induce positive aortic remodeling. However, improved RBS in more CADAV cases is still needed.

## Data Availability Statement

The original contributions presented in the study are included in the article/Supplementary Material, further inquiries can be directed to the corresponding author/s.

## Ethics Statement

The studies involving human participants were reviewed and approved by Committee on Ethics of Biomedicine, Second Military Medical University. The patients/participants provided their written informed consent to participate in this study.

## Author Contributions

YL for manuscript writing. ZL for manuscript editing. JF for figures and tables making. RF for procedures operating. JZ for funding providing and ethics approving. ZJ for overall designing. All authors contributed to the article and approved the submitted version.

## Funding

This study was supported by the National Natural Science Foundation of China (81870366, 81770476, and 82000430), the National Social Science Foundation of China (21ARK005), the Shanghai dawning plan (19SG31), and the Health Project of Zhejiang Province (2021KY673).

## Conflict of Interest

The authors declare that the research was conducted in the absence of any commercial or financial relationships that could be construed as a potential conflict of interest.

## Publisher's Note

All claims expressed in this article are solely those of the authors and do not necessarily represent those of their affiliated organizations, or those of the publisher, the editors and the reviewers. Any product that may be evaluated in this article, or claim that may be made by its manufacturer, is not guaranteed or endorsed by the publisher.

## Supplementary Material

Supplementary Figure 1 **(A)** MIP, 3D reconstruction and celiac trunk cross-section images of preoperative CT. **(B)** MIP, 3D reconstruction, and celiac trunk cross-section images of the latest follow-up CT in the same case.
