# Supplementary figures and images for: A Novel Solution for Distal Dilation of Chronic Dissection After Repair Involving Visceral Branches: The Road Block Strategy
Source: Front Cardiovasc Med. 2022 Mar 9;9:821260. doi: 10.3389/fcvm.2022.821260 (PMC8959700; doi:10.3389/fcvm.2022.821260)

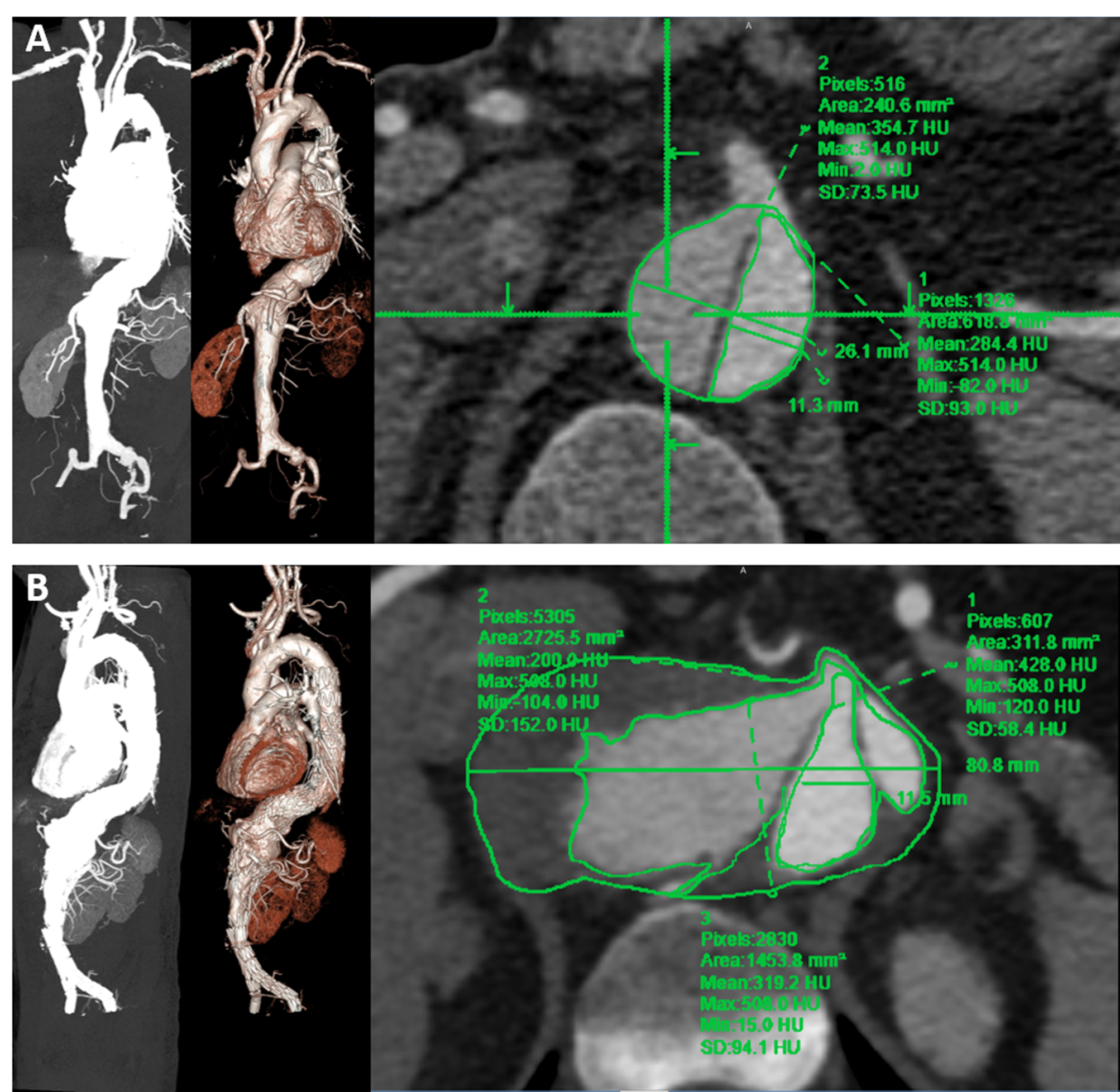

Supplement: Supplementary Figure 1 — (A) MIP, 3D reconstruction and celiac trunk cross-section images of preoperative CT. (B) MIP, 3D reconstruction, and celiac trunk cross-section images of the latest follow-up CT in the same case. [file Image_1.tif]
